# Cases of Periodical Asthma Checked by the Sulphate of Quinine

**Published:** 1848-01

**Authors:** J. C. Cain


*Cases of Periodical Asthma Checked by the Sulphate of Quinine.* By Dr. J. C. Cain, M.D. The subject of this case, a gentleman of this city, has had, during the last nine years, occasional attacks of asthma (the humid or catarrhal variety.) Within the last two or three years the attacks have become more frequent; in several of them the nervous phenomena were strongly marked, with sudden accession and equally sudden subsidence of the paroxysms. About the middle of April, of the present year, the attacks assumed a regular periodical character—coming on every Monday evening about 7 o'clock, and lasting until Friday or Saturday.

On Friday, 9th July, on the subsidence of one of the paroxysms, I commenced the exhibition of the sulph. quin. in the form of pill (which he preferred to the solution) in the dose of three grains three times daily. At the next periodical return, the attack was postponed at least twelve hours, that is, instead of coming on on Monday evening, it did not come on until after daylight on Tuesday morning. This time the paroxysm lasted only two days, and was slight when compared with those he had previously had. The sulph. quin. was suspended during the two days, and resumed on Thursday. The next Monday, the 19th, the paroxysm did not recur, and the patient felt very well. The pills were continued until the 1st of August; and up to the time I write (October 10th) he has not had a return of the disease.

*Remarks.* The foregoing case presents a striking illustration of the heroic anti-periodic properties of the sulph. quin. Its effect here was similar to that frequently observed in intermittent fever, viz: 1st, a postponement of the first paroxysm after the commencement of its administration, a diminution of its severity, and a shortening of its duration; and 2d, the prevention of the recurrence of other paroxysms. This is another instance, added to those already accumulated, which go to establish the fact that in all diseases, in which periodicity is a natural or accidental element, it is truly a specific.

*Southern Jour. Medicine and Pharmacy.*

